# Personal health records in the preclinical medical curriculum: modeling student responses in a simple educational environment utilizing Google Health

**DOI:** 10.1186/1472-6920-12-88

**Published:** 2012-09-25

**Authors:** Dimokratis A Karamanlis, Panagiotis M Tzitzis, Charalampos A Bratsas, Panagiotis D Bamidis

**Affiliations:** 1 Lab of Medical Informatics, Medical School, Aristotle University of Thessaloniki, Thessaloniki, Greece

## Abstract

**Background:**

Various problems concerning the introduction of personal health records in everyday healthcare practice are reported to be associated with physicians’ unfamiliarity with systematic means of electronically collecting health information about their patients (e.g. electronic health records - EHRs). Such barriers may further prevent the role physicians have in their patient encounters and the influence they can have in accelerating and diffusing personal health records (PHRs) to the patient community. One way to address these problems is through medical education on PHRs in the context of EHR activities within the undergraduate medical curriculum and the medical informatics courses in specific. In this paper, the development of an educational PHR activity based on Google Health is reported. Moreover, student responses on PHR’s use and utility are collected and presented. The collected responses are then modelled to relate the satisfaction level of students in such a setting to the estimation about their attitude towards PHRs in the future.

**Methods:**

The study was conducted by designing an educational scenario about PHRs, which consisted of student instruction on Google Health as a model PHR and followed the guidelines of a protocol that was constructed for this purpose. This scenario was applied to a sample of 338 first-year undergraduate medical students. A questionnaire was distributed to each one of them in order to obtain Likert-like scale data on the sample’s response with respect to the PHR that was used; the data were then further analysed descriptively and in terms of a regression analysis to model hypothesised correlations.

**Results:**

Students displayed, in general, satisfaction about the core PHR functions they used and they were optimistic about using them in the future, as they evaluated quite high up the level of their utility. The aspect they valued most in the PHR was its main role as a record-keeping tool, while their main concern was related to the negative effect their own opinion might have on the use of PHRs by patients. Finally, the estimate of their future attitudes towards PHR integration was found positively dependent of the level of PHR satisfaction that they gained through their experience (rho = 0.524, p <0.001).

**Conclusions:**

The results indicate that students support PHRs as medical record keeping helpers and perceive them as beneficial to healthcare. They also underline the importance of achieving good educational experiences in improving PHR perspectives inside such educational activities. Further research is obviously needed to establish the relative long-term effect of education to other methods of exposing future physicians to PHRs.

## Background

Personal health records (PHRs) are private and secure electronic application files through which individuals can access, manage and share their health information [1]. A PHR can include data and information entered by the individuals themselves, or data or information inputs from other sources such as health care professionals, hospital applications, laboratory or other diagnostic systems. These data may or may not be included in the health provider’s electronic health record (EHR) [2]. Many authors have discussed the potential benefits that PHR systems may bring to healthcare in the following fields, especially in the case of chronic care management: [3], patient-physician communication enhancement (secure messaging, pre-appointment questionnaires) [4-6] and public health sector(health monitoring, outbreak monitoring, linking to services and data-driven research) [7]. Thus, promoting the essence of PHRs, the wide appreciation of their need, as well as, the related research they inspire for healthcare and clinical practice, have all been targets of campaigns and white papers for the related European Commission Units as well as professional European Societies and their Alliances [8-11]. In general, interventional studies have shown that PHR use may significantly help patients engage preventive health care, like vaccinations [12] or mammography [13], or decrease patient-related problems, like medication discrepancies [14]. The importance of PHRs and eHealth and its impact especially on patient safety and risk management has also been underlined [15,16]. However, when it comes to medical conditions, results concerning the effect of PHR use on patient health quality measures (e.g. in cases of hypertension, diabetes) reveal little or no significant improvement [17,18]. However, even in the latter theoretically negative cases, it is still admitted that a mere provision of PHR on its own may be indeed limiting the impact on health quality measures, or patient empowerment/ satisfaction with care. It is thus anticipated that additional education may increase PHR use and hopefully the impact of such clinical interventions [17].

However, large-scale PHR adoption will increasingly be dependent upon the support and acceptance by physician practices [19]. Many reports and papers have recently admitted and discussed the pivotal role physicians have in educating patients during their encounters about their individual health matters [20-22], general health attitudes and behaviours [23] as well as the advantages of PHRs, their proper use and functionalities [24,25]. Such physician originated instruction and education promotes the patient involvement in the clinical workflow through increased PHR use, since it provides patients with care incentives and the reassuring feeling that record-keeping has a positive impact on their health. In general, one could describe physicians’ attitudes towards PHRs as an external, but key influence on PHR diffusion in the patient community and society, by directly affecting the so called “coefficient of innovation” according to the Bass technology diffusion model [26].

However, for one to accelerate the aforementioned physician-lead PHR diffusion, numerous problems need to be addressed and resolved. To start with, physicians are reported to present relatively low level of awareness of their patients’ PHRs [27]; moreover, they seem to be greatly concerned about the demands of time spent on a PHR examination [6,28,29]; last but not least, the lack of trust in patient-originating PHR contents cannot be overlooked [30]. One of the possible main causes of these problems might be the disturbance or alteration of the traditional patient-physician interaction model along with recent internet-centred technological advances [5,31,32], as some physicians point out [33]. In other words, this relationship, which for centuries has been the exclusive province of health care providers, has recently witnessed fundamental changes stepping into the era of free information exchange, the emergence of the collaborative or else social web (Web2.0) [34,35], and the wide availability of non-hospital centred, web-based patient health record applications. However, another more realistic cause might be the physician unfamiliarity with PHRs (or even EHRs) which in fact seems to be creating serious misconceptions about their use and importance [6], and the proper way of their incorporation into clinical practice. On the other hand, if the use of PHRs is merely viewed from the perspective of being beneficial to patients only, then one might be lead to a serious concern and contradiction regarding the expected physician support [32].

To this extent, increasing physician exposure to PHRs (mainly through education), may foster support for their use in practice, while smoothing the pathway for incorporating electronic PHRs in the healthcare system [6]. As a result, the research and practice agenda on PHR adoption and the prospect of positive attitude change certainly passes through medical education [36,37]. To this extent, and according to the recommendations of the International Medical Informatics Association (IMIA) Working Group on Education in Biomedical and Health Informatics [38], physicians, as information and communication technology users, need to acquire an intermediate level of knowledge on systematic information processing in healthcare, as well as, the ability to communicate electronically. Curricula about PHRs need to be developed in the context of medical informatics courses, based on suggestions about EHR education [39], and should exist within every single level of their education (medical schools, internships, residencies, fellowships, and in services), if possible [40]. Such curricula should provide comprehensive education about PHR use, its place in the health information exchange movement [19] and the benefits physicians obtain by using them [4]. They should also focus on allowing physicians to acquire skills and knowledge on how to educate their patients about PHRs, so that they become capable of encouraging them to enter the information accurately, to trust that information appropriately and to interpret the information they are receiving from the physician’s EHR [32].

Along the above lines, the course of Medical Informatics at the undergraduate Medical Curriculum of the Aristotle University of Thessaloniki (AUTH), Greece, had always contained elements of EHRs and PHRs [41]. This course has recently been expanded to allow for new teaching approaches and the incorporation of new technologies [42-44] as well as the provision of knowledge and practice on contemporary, widely available and used PHR systems like that offered by giants like Google [45]. In this sense, the aim of this paper is twofold: first, to report on the development of an educational PHR activity based on Google Health in the aforementioned undergraduate medical informatics module and to collect the students’ views on PHR functions and their concerns towards their use; second, to use these responses in order to try and relate the satisfaction level of students in such a setting with their likely attitude towards using PHRs in the future, thereby creating a model for improving positive attitudes by facilitating a rich experience of early PHR use and adoption.

## Methods

The study was conducted by designing a controlled educational scenario about PHRs and applying it to a cohort of undergraduate medical students. At the end of the educational session, a purpose-built questionnaire was distributed to every student receiving this education, in an effort to obtain data on the student responses and attitudes against the PHR they used; these data were then statistically analysed and modelled in order to predict future attitudes and intentions for PHR use. An approval from the Ethical Committee of the Medical School of the Aristotle University of Thessaloniki, Greece, was granted for this study (ref No. A5726).

### Settings and subjects

The sample consisted of 1^st^ year cohort of undergraduate students of the Medical and Dental Schools of the AUTH, who participated in a single two-hour laboratory session, which was structured on the basis of the educational PHR scenario. This lab session was part of the Medical Informatics I course of the academic year 2010–2011 and was offered in November 2010 (it was the 5^th^ in a series of six (6) lab sessions on acquiring practical medical informatics skills), and accompanied by the theoretical EHR/PHR lecture given (along the traditional way) in the amphitheatre. Thus, presumably, each student had acquired theoretical knowledge about EHR/PHR systems before committing to the lab activity. We assume that the sample is uniform with respect to the background obtained by students (medicine or dentistry), because, at the time this lab was taught, all students had gained similar learning experiences by following the teaching schedule of basic medical sciences; thus, from now on, the sample will be merely called as “medical students”.

### Educational scenario

The educational scenario consisted of students obtaining instructions on Google Health as a model of a PHR, which followed the guidelines of a protocol that was constructed on this purpose.

The educational scenario was chosen to be patient-oriented, i.e. considering and facing students as individual PHR users. As the course was offered to first-year students in a preclinical context, it is imperative it could not be considered to support clinical decision making. In addition, the scenario could not assume that would foster skills they would directly require in the future; nonetheless, it can be confidently stated that the scenario was deemed appropriate and possibly useful as a first practical encounter with PHRs and, more importantly, as a good example of how they could aim to educate their (future) patients to use this or similar system in the following years.

It also had to be based on the context of the other four (4) preceding lab scenarios, which the students followed weekly before the PHR one. As a result, the educational scenario was applied to thirty three (33) small groups of students (each of about ten (10) to fifteen (15) students), which followed, individually (i.e. not as a group work) the two (2) hour- long laboratory session. The protocol was facilitated by two (2) instructors in each lab session, who were responsible for one group of students. The instructors, mostly graduate or research students, were all associates/members of the Lab of Medical Informatics at AUTH. The protocol, as well as, all accompanying teaching material was provided to them in an “Educating the Educators” mode, at least one (1) week before them administering the actual lab session. Needless to mention of course, that the drafting of all this educational material (student handouts as well as the instructions and aids for educators underwent a series of revision rounds so as to minimise any likely drawbacks).

In the scenario, Google Health, one of the two major PHR service Web providers (the other one being Microsoft HealthVault [46]), was chosen as a model PHR system for this sample, due to the relative simplicity of its architecture (compared to Microsoft HealthVault [47]) and the minimal list of prerequisites needed. The only requirements which had to be met were the existence of an Internet connection and a Gmail account for every computer unit that was to be utilised. Thus, the scenario to be run was relatively simple even for first-year medical students, as the majority of them are, nowadays, Web-literate at the basic level [48]. The version of Google Health used for both the drafting of the protocol and the education of students was the one available after September 15, 2010. Because it was to be used as a model PHR, we only chose core PHR functionalities to be presented, without any third-party applications that Google Health might have been cooperating with at that time.

The final protocol included three parts:

• An oral, slideshow-based, presentation was made by the instructor on PHRs in the context of EHRs and, then, Google Health. The EHR definition was given [49], followed by the PHR definition [1] and the most important characteristics which a PHR should incorporate [50]. Information on Google Health’s functionalities and privacy policy [51] were also provided through the same means. This procedure was a necessary step in order to assure that every student has a minimum, basic background knowledge concerning the aims of the very lab session and the objectives they were attempting to achieve, while at same time forming a PHR reference (control) to be compared to Google Health during the survey that was following (see below).

• While every student was connected with Google Health through their computer, the instruction was given through a wall projection screen on about 7 topics/tasks, namely: entering and managing health data (Profile Options), finding information about them (Health Topics), sharing them with other Google Health accounts (Sharing), spotting-interpreting drug interaction warnings (Drug Interactions), managing medical contacts (Medical Contacts), searching for health professionals (Search for a Doctor) and managing multiple profiles (Add Another Profile). The students had to follow the instructors’ actions on every subject.

• The students were graded according to their responses in a simple exercise, which tested the abilities acquired upon entering and managing health data, managing multiple profiles and spotting-interpreting drug interactions. The exercise was based on a real life scenario, which students were supposed to analyze and follow as PHR users (or else citizens/patients). A hand-out about a patient’s short health story was distributed to them. At first, they had to retrieve valuable medical information, as much as possible, through it. Then, they had to create another profile besides their own, fill-in that information and interpret the drug interactions that appeared between either medications or medications and conditions.

Before going any further, let us make a second assumption about the uniformity of the scenario application throughout the different student groups. This assumption is based on the fact that, despite the difference between the instructors of each group, there was the common protocol for their actions, which presumable was followed in a more or less uniform way, as it was accompanied with step-wise instructions in the “educator’s aid” set.

### Survey design and administration

The questionnaire was administered to the students, right after the end of the educational scenario, in the form of if an online survey created using the LimeSurvey open source software utility. After suitable piloting and review rounds, it was finally composed of fourteen (14) close-ended, obligatory questions and one (1) open-ended mandatory question. Out of the closed type, 12 were Likert-type, containing five (5) ordinal answers mapped to a 1–5 integer scale. Some of them required the completion of more than one fields. This paper focuses on the components of the survey that are related to the personal health record and its related aims.

Question themes were organised into two groups within the questionnaire, one about user satisfaction of Google Health as a PHR application and one about their future expectations on Google Health use. This distinction was made so as to serve in the last part of the study which deals with the relation between aforementioned themes, thereby attempting to obtain a model of user intention.

In the case of their current satisfaction, students were asked to grade some items based on the experience they had gained during the educational scenario. The questions included (group I) were about:

a) the level of satisfaction of Google Health as an application, by commenting on 5 characteristics, namely: Ease of Use, Speed, Visual Appeal, Privacy and Security Issues, overall Features and Capabilities. This question was based on the questionnaire Google used to gather information for their own product [52].

b) the level of satisfaction of Google Health as a PHR, by commenting on the seven (7) functions that were presented to them during the instruction, namely: Profile Options, Health Topics, Sharing, Drug Interactions, Medical Contacts, Search for a Doctor, Add Another Profile.

c) the level of their concern on using Google Health when considering three (3) distinct subjects: Physician’s Opinion, Accessibility, Gathering Anonymous Statistical Data.

There was also an open-type question at the end of the questionnaire, in which students were asked to comment on the lab session design itself and suggest ways of improving it.

In case of future expectations, students were asked to grade some items based on their thoughts about what their future self as a physician would be. Thus, the questions included (group II) referred to the students’ future attitude towards PHRs in general and were, therefore, about:

a) the likelihood of proposing Google Health to their patients if the latter did not use any electronic PHR at all.

b) the usefulness and utility that a Google Health patient account would have for to the delivery of healthcare with regards to each of the following five (5) aspects: maintaining an up-To-Date Medical Record, Doctor-Patient Communication, Emergency Situations, Accessibility to Patients, Observing Health Trends.

c) and finally, the effect that Google Health would have on their clinical practice, if their patients began using it.

Instead of referring to PHRs in general, it was considered more proper that question subjects were focused only around Google Health, as this was the one that students interacted with and were trained in. However, the results were attempted to be generalised into PHRs, because this product was just used as a model of a common Web-based PHR; this was emphasised during the theoretical part of the lab session.

### Data analysis

The questions chosen to be of importance individually were those about satisfaction of Google Health as a PHR, the level of concern on using it, the utility to the future healthcare. Results were aggregated and then stratified by the type of the student curriculum (medicine, dentistry); in order to check our hypothesis concerning the sample’s uniformity in the two different groups, the Mann–Whitney U test was used to verify its validity. Only frequency-based descriptive analyses were conducted to evaluate the collected students’ response for every item under the suggestions of Jamieson [53] on handling Likert-type ordinal data. Moreover, a classification of the items in each question was made, based on the proportion of the number of answers corresponding to the two highest question ranks to the total number of answers. The significance level was set at p < 0.05 for all statistical comparisons. Student comments from the open-ended question were also collected and evaluated manually, according to the semantics of their content.

To obtain a more general view about the students’ views, we constructed two questionnaire-based indices for each student, with values ranging in the [0,1] interval, which are the transformed averages ($\left(m-1\right)/4$, where *m* is the average) of their answer scores, in specific questions (different for each index). This was done under an important hypothesis, which we have to introduce at this point. To get the values of the indices for each student, we assume that their answers were mapped to an interval scale. Each index was given a meaning according to these questions and was characterized as a new observable about the students on PHRs. More specifically, the two indices were:

• Experience index (EI) which corresponded to student answers to questions concerning their level of satisfaction of Google Health in the educational scenario. It included the answers from every field of satisfaction of the product as an application and as a PHR (group I subgroups: (a) with five and (b) with seven items). This quantification may be interpreted as the quality of the experience gained by the student during the interaction with a PHR in this certain educational environment.

• Future doctor index (FDI) which corresponded to student answers to all questions that considered them as a future health professional (group II subgroups: (a) with a single, (b) with five and (c) with a single item). Such questions were those referring to Google Health’s effect on their future clinical practice and their likely attitude towards the PHR diffusion. An easily understood and, later on, applicable interpretation of FDI would be that of an estimate of a PHR’s integration into a student’s future clinical practice. By integration, we hereby define, a physician’s willingness to cooperate with patients already using a PHR or to urge others not owing or using a PHR, to start using one. To be more specific, let us make provide some quantitative insight of the FDI values. Let’s consider the total number of patients (***n***) that will visit a physician for the first time during his clinical practice. We define integration (***p***) as the proportion of the number of PHR-keeping patients that the physician will cooperate with and of non-PHR-keeping patients to whom the physician will suggest (and educate, maybe) PHR use (***k***) to the total number of patients (p=k/n). As a result, the estimate that we made can be rewritten as: FDI=p_._

In order to check the correlation between these two indices, the measure selected was Spearman’s rho. Correlations of the two different student groups (medicine, dentistry) were also calculated and compared by using the Fisher’s z transformation to their values first.

## Results

There were 338 questionnaire entries by 261 (77.2%) medicine students and 77 (22.8%) dentistry students. There were no missing responses, except one (1) in the Observing Health Trends field of the question about Google Health’s utility in healthcare. This data element was left missing.

The results of the evaluation of Google Health functions by students are shown in Table 1. Drug interactions, Profile Options and Add Another Profile features were the ones which students rated higher than the others, as their percents of high to maximum answers were the largest compared to the other functions, 81%, 76% and 70% respectively. Students seemed less satisfied by the Search for a Doctor feature, in which more than the half (about 58%) reported minimum to medium satisfaction, with the most common answer referring to the medium level. None of the features significantly differed between the averages of groups of medicine and dentistry students, except the “Health Topics” (p = 0.033), which the medicine students evaluated higher.

**Table 1:** Student satisfaction levels about Google Health functions

**Functions**	**Level of satisfaction**						
	**Minimum (1)**	**Low (2)**	**Medium (3)**	**High (4)**	**Maximum (5)**	**Median**	**Mode**
Drug Interactions	2(0.6%)	10(3%)	51(15.1%)	153(45.3%)	122(36.1%)	4	4
Profile Options	3(0.9%)	17(5%)	60(17.8%)	161(47.6%)	97(28.7%)	4	4
Add Another Profile	4(1.2%)	24(7.1%)	73(21.6%)	117(34.6%)	120(35.5%)	4	5
Health Topics	7(2.1%)	18(5.3%)	88(26%)	137(40.5%)	88(26%)	4	4
Medical Contacts	8(2.4%)	32(9.5%)	99(29.3%)	129(38.2%)	70(20.7%)	4	4
Sharing	9(2.7%)	43(12.7%)	114(33.7%)	118(34.9%)	54(16%)	4	4
Search for a Doctor	18(5.3%)	64(18.9%)	113(33.4%)	104(30.8%)	39(11.5%)	4	3

Student answers to the question: “What is the level of your satisfaction after using Google Health in each of its following functions?”.

Generally, students showed their concern about the patients’ use of Google Health in all aspects (Table 2). The level of concern was relatively higher in the case of the Physician’s Opinion (72% of student answers were in the high to maximum choices), followed by the cases of Gathering Anonymous Statistical Data (65% high to maximum choices) and Accessibility (64% high to maximum choices). For any of these three factors no statistically significant differences between averages of groups of medicine and dentistry students were observed.

**Table 2:** Student level of concern about the use of Google Health

**Aspects**	**Level of concern**						
	**Minimum (1)**	**Low (2)**	**Medium (3)**	**High (4)**	**Maximum (5)**	**Median**	**Mode**
Physician's Opinion	10(3%)	21(6.2%)	65(19.2%)	123(36.4%)	119(35.2%)	4	4
Gathering Anonymous Statistical Data	12(3.6%)	26(7.7%)	79(23.4%)	108(32%)	113(33.4%)	4	5
Accessibility	11(3.3%)	27(8%)	83(24.6%)	136(40.2%)	81(24%)	4	4

Student answers to the question: “What is the level of your concern as a patient about the use of Google Health in the following aspects?”.

Pretending to be future health professionals, students evaluated the utility of Google Health in various fields of clinical practice (Table 3). It appears that they found utility/usefulness in every aspect described lying somewhere between the medium and maximum level (the mean percentage of the high to maximum answers in every field was around 66%). The utility was rated higher in the cases of Up-To-Date Medical Record (the mode was the maximum level and the high to maximum answers exceeded 77%) and Accessibility To Patients (the mode was shared between high and maximum levels, with their percentage of answers being close to 70%), while it seems to have been rated the lowest of all in the case of Patient-Physician Communication (percentage of minimum to medium levels was approximately 60%). For any of these fields there was no statistically significant difference between averages of groups of medicine and dentistry students except for the utility in Emergency Situations, which dentistry students evaluated higher (p = 0.039).

**Table 3:** Student view on the utility level of Google Health in certain aspects

**Aspects**	**Level of utility**						
	**Minimum (1)**	**Low (2)**	**Medium (3)**	**High (4)**	**Maximum (5)**	**Median**	**Mode**
Up-To-Date Medical Record	8(2.4%)	16(4.7%)	53(15.7%)	119(35.2%)	142(42%)	4	5
Accessibility To Patients	5(1.5%)	20(5.9%)	75(22.2%)	119(35.2%)	119(35.2%)	4	4*
Observing Health Trends	7(2.1%)	25(7.4%)	81(24%)	127(37.6%)	97(28.7%)	4	4
Emergency Situations	17(5%)	41(12.1%)	76(22.5%)	87(25.7%)	117(34.6%)	4	5
Patient-Physician Communication	11(3.3%)	39(11.5%)	86(25.4%)	110(32.5%)	92(27.2%)	4	4

Student answers to the question: “As a future health professional, of how much utility would it be for healthcare the fact that a patient has a frequently updated Google Health profile in the following aspects?”.

* The mode is 4 or 5.

There were too few written comments in the open question for a serious thematic analysis; however, several students commented on their satisfaction of the lab session. Table 4 outlines selected student comments regarding the lab session on Google Health. Students valued learning to work in a PHR environment – "…the knowledge that we’ll really need in the future!" They also had concerns about privacy issues linked with PHRs (Table 4) – "patients’ personal data should be strictly protected" and made suggestions about the way the lab session should be in the future– “Electronic Health Records should also be taught…”.

**Table 4:** Selected student comments


1 “Electronic Health Records should also be taught, not just Google Health.”
2 “The student should be able to freely create a profile with the information he/she wants and then present it to the instructor”
3 “Patients’ personal data should be strictly protected.”
4 “It’s useful enough for now, but it’ll be more in the years to come.”
5 “Learning about Google Health was very useful! Maybe it's the knowledge that we’ll really need in the future!”

Selected student responses in the open-type question of the questionnaire, when they were asked to comment on their experience of the lesson and their opinion on how it could be improved.

**Figure 1:**
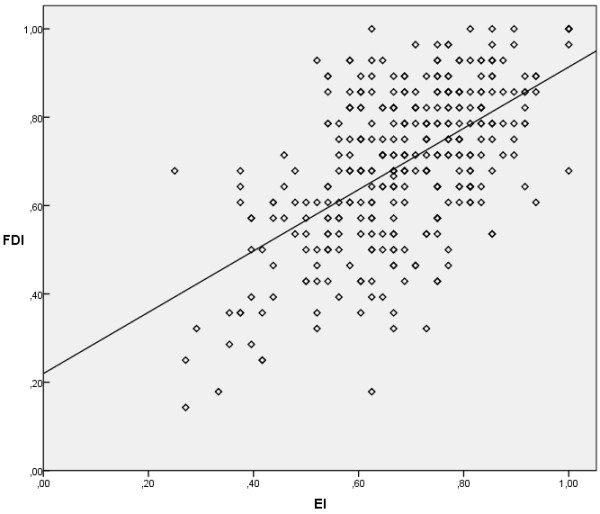
**EI-FDI scatter plot.** Experience index (EI) and future doctor index (FDI) in (EI, FDI) pairs presented as circles in a scatter plot. Because the values of the questions which produced them were fixed, so are their values and, as a result, there are less circles than the sample size. To illustrate the simple case of the linear relationship between the indices, the least squares line with equation FDI = 0.694EI + 0.22 and R^2^ = 0.33 is presented in the figure.

The arithmetic means and standard deviations of both EI and FDI were m = 0.68, SD = 0.15 and m = 0.69, SD = 0.18, respectively. Both the values of EI (p = 0.480) and FDI (p = 0.129) do not appear to differ significantly between groups of medicine and dentistry students. The nonparametric Spearman's test revealed a positive dependency between the two indices (rho = 0.524, p <0.001), suggesting that the increase in one’s value implies a corresponding increase of the other. Figure 1 is the scatter plot of (EI,FDI) pairs and illustrates the least squares straight line (FDI = 0.694EI + 0.22, R^2^ = 0.33), in case these pairs are supposed to be linearly related. Spearman’s rho differed significantly between the two groups of students (p = 0.015), with bigger rhos for medicine (rho = 0.556, p <0.001) compared to dentistry (rho = 0.365, p = 0.001) students.

## Discussion

This paper described the organisation of a prototype preclinical patient-oriented educational scenario about PHRs in a medical informatics course. Students displayed, in general, satisfaction about the PHR they used and they were optimistic about using it in the future. They have valued most PHR’s main role as a medical record, but they have expressed some concerns about the PHR use by patients as a mere result of the opinion of the responsible physician towards its use. The simple prediction of their future attitudes towards PHR integration was found to be dependent on the level of PHR satisfaction that they gained through the educational encounter and experience gained, thereby indicating the pivotal role of good educational experiences and contemporary topic lab designs may play in improving PHR perspectives.

One can easily observe that the three features of Google Health which were rated higher were those which were assessed during the exercise part of the scenario. The fact that only some (and not all) features were part of the exercise may have led students to a slight overestimation of their significance with respect to the rest. The importance of assessing students in such an educational scenario is evident in this case. Assessment can be really considered as a tool to further improve the experience of students with the functions of a PHR in general, and was considered necessary as it may provide further means to emphasise the importance of using PHRs in clinical practice. Speaking about the importance of the Drug Interactions feature in PHRs, students seemed to like the way that it makes the patients more active when it is used (one should recall that the “patient-centric” scenario presented students with the chance to experience it as patients). Furthermore, as expected, the satisfaction concerning the Search for a Doctor feature was lower compared to other features, as this one is not properly functional internationally in the Google Health environment (the course was offered in Greek). Thus, the results of the searches were not as accurate when students searched for physicians outside the USA. Although it’s a critical feature for PHRs, as it connects patients to their physicians (PHRs to EHRs in general), the Sharing feature in Google Health corresponded to a relatively large percent (about 49%) of minimum-medium responses. A possible reason is that students found the presentation of this feature uninteresting, as the data shared were the same that they had to complete when training for the Profile Options feature. The statistically significant lower mean of dentistry students on their satisfaction of the Health Topics feature is probably due to the absence of presenting highly specific information on dental issues within the overall protocol.

The fact that the Physician’s Opinion factor concerns students the most in using Google Health, is probably due to their identity as future physicians. It also underlines the importance of having physicians with positive attitudes towards PHRs to facilitate PHR diffusion. It can also be noticed that Accessibility to the PHR (ability to operate a computer, familiarity with the Internet) is a factor which is particularly important to the use of PHRs. This is consistent with other results describing the reduced contact with PHRs that people with little computer skills have in relation to others [54]. However, the fact that it was ranked lower than the other two seems to correspond to the increased familiarity of the new generation of health professionals (first-years) with computers and the Internet [48], as they pretended themselves to be the patients in the scenario herein. Gathering Anonymous Statistical Data from patients seems to be a major concern for them also, which presents a conflict with the Observing Health Trends aspect of the future utility of PHRs (they presented almost the same high to maximum answer percentages, around 65%). This conflict cannot, of course, be attributed to possible threats to a patient’s privacy, as the data collection was defined as anonymous. One plausible cause is that the students are concerned about the use of these data mostly for commercial profit, with less emphasis on healthcare improvements or scientific empowerment. The fact that the question referred to Google Health may be crucial here, because it may have directed student concerns to the importance of these reasons.

With respect to the likely future use of PHRs by patients, seemingly all areas described in the questionnaire provide statistical evidence about the future clinical practice of students; this is revealed by both the data and the students’ (qualitative) comments. The utility on the Up-To-Date Medical Record was rated high compared to all other aspects, which is consistent with the high rank that the Profile Options feature achieved. This observation is also consistent with one of the key advantages that can be derived from an interconnection between PHRs and the physician’s EHR system. The PHR utility in Physician-Patient Communication was rated lower than anything else, which outlines one of the major problems described about physicians and PHRs: change in the traditional relationship between them and the patients. It’s evident even in first-year students [55], that the physician’s role is such that it only supports communication in a certain way, so transferring this link into the Web through PHRs may be perceived as a danger of eliminating it.

The positive dependence between the two indices partially verifies the ideas about the usefulness of the undergraduate education of health professionals in the diffusion of PHR to the community of patients, although the experience that students gained was through a patient-oriented medical scenario. Achieving proper experience in such a scenario may improve the attitude of students towards PHRs, reflected by the FDI, so when they encounter them later in their education or clinical practice, they will be more urged to use them, as they will be suitably aware of the subject. The difference of EI-FDI dependence between the two student groups can be due to the fact that the scenario was mainly designed for medical students, as the topics discussed concerned mostly them. Although the simple linear model may not be the best fit for our dataset, it can provide useful insights on the scenario. Firstly, if EI = 0 was to be interpreted as no contact with the scenario, the level of FDI would be 0.22, which reflects the estimate of the PHR integration (approximately to a level of 20% of the total patients) without the existence of the scenario. Secondly, a student’s maximum satisfaction (EI = 1) corresponds to FDI = 0.914, which leads us to another conclusion: the education-attributed integration is 0.694, which corresponds to a potential 0-70% increase of the patients that will be affected by the physician’s positive attitude towards PHRs.

Finally, the current piece of work is by no means moderated by Google’s recent announcement their retirement from the PHR Web service (note that it was reported that Google Health “is not having the broad impact that we hoped it would” [56]). In fact, this underlines the emerging importance of physician education on PHRs, as having more PHR-educated physicians would be crucial in creating large-scale studies about the features that are most valued by them in a PHR product. This would attract major players in Web market to create refined and easy-to-use PHR products that emphasize those features.

### Limitations

Since no stratification of students was conducted with respect to their PHR background or, generally, electronic record keeping familiarity, this might introduce some bias in the answers of the questionnaire, as our sample might have displayed generally better (or worse) attitude towards PHRs relative to a sample that had no members with previous PHR interaction. Another factor that introduces positive bias to the answers of our sample is the fact that the questionnaire was completed inside the educational activity, as students might have considered it as a part of a common lesson evaluation.

The scenario through which the EI and FDI values were acquired was patient-oriented. The experience that medical students will get through a PHR scenario may be one of a different educational perspective, so one cannot really generalise the dependence in their values well. The fact that the students were first-years presents some problems when it comes to the part of the questionnaire related to their future expectations, as their judgement on that is not based on real experience, but mere hypothetical thinking. As a result, FDI, which is directly related to these questions, suffers from interpretation bias, as it is just a measure of the estimate of students’ future attitude. The actual level of the PHR integration probability may differ, if we were to study the long-term students’ behaviour, after they had obtained enough clinical experience (e.g. during the last two years of their studies). These facts may undermine any possible practical importance that the results would have on further applications.

### Implications for Future Research

It is suggested that medical informatics courses that require sufficient medical background, such as those on EHRs and PHRs, should be placed towards the clinical part of the medical curriculum, to facilitate the use of the items learned [57]. Preclinical introductory courses about these items can also exist to provide information literacy and help students develop practical skills [58]. Considering all these, educational activities based on PHRs should be formally described, so that medical schools can easily integrate them in their curricula. Interventional studies comparing “educated” groups against “uneducated” groups of students could also be organised, so as to fully determine if the presence of an educational scenario were to improve student attitudes towards PHRs.

Studying the results of a similar physician-oriented educational process (teaching different skills to students, such as interconnecting an EHR system with PHRs) would make apparent if undergraduate scenarios in PHRs have a direct effect to better future PHR attitudes. A comparison between the results of the two educational approaches afore-described, i.e. patient or physician-oriented, would also be made possible in that case. It can be stated that, besides the serious limitations that go with them, the first years’ results may be used in a later study concerning the temporal evolution of an intervention’s effect on the sample (by us or by others who want to address the same subject).

Finally, there are obviously more PHR products which include all the characteristics needed in a similar educational approach (Indivo [59,60], Microsoft Health Vault [61]). The results from the application of a similar scenario, which would use a different PHR, could be compared to the present ones. Moreover, an educational scenario that would incorporate data from multiple PHRs could be created, thereby making possible the study of the effect of PHR product on the students’ opinions.

In a “self-caring society”, patients would have liked to take full part in deciding about their treatment in a symmetric and negotiated relationship with healthcare professionals; for the later to be able to react on their patients’ empowerment they need to be educated and familiar with PHR systems that patients are likely to interact with. For example, patients joining the PatientsLikeMe community go online to (not only discuss health and daily living) but to share detailed health data. Strictly speaking, of course, PatientsLikeMe is not a PHR system, but rather a “shared” online platform where patients “share structured information about symptoms, treatments, and outcomes, view individual and aggregated reports of these data, and discuss health and garner support on forums and through private messages”. Members of PatientsLikeMe offer one another support based on their own personal experience and advise each other on both medical issues and how to improve day-to-day life [62].

Recent studies with such PHR resembling systems, provide evidence that patient-reported data and outcomes, offer a unique real-time approach to understand utilization and performance of treatments across many conditions and potentially identify targets for treatments [63].

But since PatientsLikeMe is something between a classical PHR and an information searching platform, one could indirectly assume that the effects web searching and information sharing technologies bring about with respect to the patient empowerment and the change of doctor-patient relationships [64-66] are related to our study. This makes stronger the need to keep a record of how professionals are educated with respect to these systems and how this education could be potentially modeled to enable future optimization of the societal benefits outweighing potential technology threats.

## Conclusions

To conclude, this paper had a dual aim: first, to report on the development of an educational PHR activity based on Google Health and related issues and concerns towards its use and second, to use and model these responses in order to try and relate the satisfaction level with the future professional intentions. We have shown that the experience of students by implementing an educational scenario on issues related to PHR is significantly related to their estimate about the future integration of such a system into their clinical practice. If one was to ignore the serious limitations that our sample goes with and assume that a direct correlation between the current estimation of students with the real prospects of acceptance of a PHR in their clinical practice exists, it seems reasonable to allow for their inclusion in the medical informatics course of the undergraduate medical curriculum. A more complete educational experience will lead to a better cumulative estimate, leading to the optimum future contact in the physician-patient relationship, which will, probably, accelerate PHR diffusion.

## Competing interests

The authors declare that they have no competing interests.

## Authors’ contributions

DAK led on design, statistical analysis and interpretation of data and drafted the manuscript. PMT contributed to interpretation of data, the revising of the different paper sections and assisted in drafting the manuscript. CAB was responsible for guiding the design and implementation of the lab, as well as the educating the educators sessions; he also assisted in the conception and participated in the statistical analysis too. PDB perceived the initial concept of this educational and research attempt. He contributed to the design, revision of both the original and the resubmitted article and critically appraising the content. All authors have read and approved the final version of the article submitted.

## Pre-publication history

The pre-publication history for this paper can be accessed here:

http://www.biomedcentral.com/1472-6920/12/88/prepub
